# Importance of visual and proprioceptive inputs for maintaining balance in patients with chronic non-specific neck pain: A cross-sectional study

**DOI:** 10.1371/journal.pone.0340633

**Published:** 2026-03-05

**Authors:** Wenwen Wei, Wenxiang Li, Yixin Wang, Shuohan Zhang, Guoqiang Fan, Yiwen Bai

**Affiliations:** 1 School of Rehabilitation Science, Shanghai University of Traditional Chinese Medicine, Shanghai, China; 2 Department of Rehabilitation, Huashan Hospital, Fudan University, Shanghai, China; 3 Department of Sports, Shanghai University of Traditional Chinese Medicine, Shanghai, China; 4 School of Rehabilitation Science, Shanghai University of Sport, Shanghai, China; Ordu University, TÜRKIYE

## Abstract

**Objective:**

The aim of this study was to investigate the differences in muscle activation among patients with chronic non-specific neck pain (CNSNP) when performing resistance movements in different sensory inputs in a sitting position, using surface electromyography (sEMG) as an indicator.

**Materials and methods:**

A total of 39 participants with CNSNP were recruited. sEMG recordings of the respective cervical muscles were measured for participants seated on four surface categories: eyes-closed-dynamic-surface, eyes-open-dynamic-surface, eyes-closed-stable-surface and eyes-open-stable-surface during neck extension, flexion, left and right lateral flexion, left and right rotation. Muscle activities in each situation were conducted thrice times and normalized as the percentage of maximum voluntary contraction (%MVC).

**Results:**

Two-factor repeated measures analysis of variance (ANOVA) of the study data showed that the MVC% of respective muscles when seated on a dynamic surface was significantly lower compared with that recorded when seated on a stable surface, for the movements of extension (p = 0.012), flexion(p < 0.001), left lateral flexion(p = 0.001), right lateral flexion(p < 0.001) and left rotation(p = 0.0163). Simple effect analysis of the study data showed that the MVC% recorded for right rotation when seated on an eyes-closed-dynamic surface was lower than those recorded in the other three surface categories.

**Conclusion:**

In this study, different sEMG activities of cervical muscles were identified under different sensory inputs in patients with CNSNP. This sheds light on the sensory integration and compensatory mechanisms in patients with CNSNP, and helps to guide their clinical management.

## Introduction

Chronic non-specific neck pain (CNSNP) refers to neck pain lasting more than 3 months without a specific pathological cause, which is the fourth leading cause of disability [1]. It may associate with sleep disorders, smoking, trauma, obesity and psychological factors [2–5]. However, the main cause may be the overstraining of neck muscles [6], which is of vital importance in maintaining neck stability and has been affecting young populations in recent years [1,2].

Cervical muscles play a critical role in sensorimotor function, having abundant cervical mechanoreceptors which integrate multisensory afferent inputs, including proprioception, vestibular, visual and somatosensory information [7]. Sensory impairment in the neck is one of the main issues of patients with neck pain, leading to the impaired sensorimotor control of the neck [8,9]. In the sensorimotor system’s three subsystems, the vestibular subsystem provides information about the head’s position relative to gravity. The visual subsystem uses external cues to identify the body’s position relative to the surrounding environment [9], and the somatosensory subsystem encompasses all mechanoreceptive information arising from the periphery, including proprioception from the neck and somatosensation from the rest of the body, especially the foot [10,11]. Sensorimotor control in the neck involves the central integration and processing of all incoming sensory information to execute motor programs through the neck muscles, aiding in maintaining head position, balance and stability of the neck joints [12]. An experiment concerning balance changes in elderly individuals with neck pain suggested that patients with neck pain may exhibit sensory reweighting and sensory inputs from these three systems are recalibrated [13]. This process includes increased reliance on proprioceptive inputs from the lower limbs to compensate for deficits in neck proprioception.

Surface electromyography (sEMG) can detect electrical activity in muscles and has thus been used in many fields, such as neurophysiology, sports and physical therapy [14]. It facilitates the evaluation of muscle activity during movements and can be used in measuring the activity of cervical muscles [15]. Cervical muscles in patients with non-specific neck pain show a considerably higher degree of activity than those in healthy adults during isometric contraction in the neutral head position [16]. This difference is primarily due to protective mechanisms developed by patients with neck pain in response to pain. This increase in activity shifts activity from deeper muscles to superficial muscles, affecting proprioception [3,17].

Several studies about have examined in patients with CNSNP [18–22]. Some of these studies focused on the older [18,21], demonstrating a poorer postural stability in CNSNP individuals in comparison with healthy individuals. Researches indicated reduced postural stability in patients with CNSNP was associated with catastrophic thinking [18], neck torsion maneuver [21], longus colli cross-sectional area (CSA) [22]. Others [19,20] focused on center of pressure (COP) as an outcome measure in young adults to prevent the effects of aging, which found the similar results. Specifically, SAADAT M et al [20]’s study explored dynamic postural stability in young patients with CNSNP, finding that these patients exhibited greater trunk sway during dynamic testing whether with eyes open or closed. Nevertheless, to the best of our knowledge, there are no investigations that have examined sitting dynamic postural stability in patients with chronic neck pain. And the impairment and compensatory relationships among the three subsystems of sensorimotor system have not been explored so far, too.

Therefore, we focused on the 18–40-year-old age group to prevent the expected effects of degenerative changes that may occur in older adults and compared the degree of activation of neck muscles during resisted neck movements under four different conditions. This study aimed to investigate the differential effects of visual and support-surface somatosensory inputs (which primarily interferes with lower limb proprioceptive input) on cervical muscle activation in young adults with CNSNP. It was hypothesized that cervical muscle activation would be significantly lower under dynamic-surface compared to stable-surface and the removal of visual input would have little effect on cervical muscle activation, indicating a greater reliance on somatosensory inputs from the lower limbs for postural stability in this young population.

## Materials and methods

### Study design

A cross-sectional study was conducted to assess the recruitment of neck muscles in six directions (e.g., flexion, extension, bilateral lateral flexion and bilateral rotation) of neck movements under different sensory conditions. Conditions depend on the availability of visual and somatosensory inputs from the lower limbs. A priori power analysis was conducted using G*Power 3.1 to determine the minimum sample size required to detect meaningful effects in our 2 × 2 repeated-measures design. With the following parameters: effect size f = 0.25, α = 0.05, power = 0.80, which obtained a sample size of 39 subjects with 30% drop. The ethics committee of the Shanghai University of Sports approved the study (ethics approval number: 102772023RT079; date of approval: 03 July 2023).

### Participants

We recruited participants by pasting posters at Shanghai University of Traditional Chinese Medicine, Shanghai University of Sports and associated communities From June 2024 to December 2024. A total of 39 target participants (14 males and 25 females) were included in the experiment. Before each participant was accepted, an assistant checked whether they met the requirements. The inclusion and exclusion criteria were as follows:

Inclusion criteria: participants were 18–40 years old and had neck pain for more than 3 months and Numerical Rating Scale (NRS) scores of 3–8. (NRS is a self-report measure used to quantify a patient’s subjective intensity of pain from 0 (“no pain”) to 10 (“the worst pain imaginable”)).

Exclusion criteria: participants who had a history of neck trauma, surgery, tumours, injury or operation, in the cervical spine and rheumatism of the neck or shoulder.

Participants who met the inclusion criteria were informed about the purpose and contents of the study. They provided written consent. Before the start of the sEMG test, the participants filled in the questionnaire, providing information, including height, weight, NRS, Neck Disability Scale (NDI) and other scales. NDI is used to assess how much a patient’s neck pain has affected their ability to manage everyday activities. These questionnaires solely used for descriptive analysis.

### Procedure

Two muscles were measured bilaterally through sEMG: sternocleidomastoid (SCM) and splenius capitis (SPL) [23]. The skin areas where the electrodes would be placed were shaved with a razor and sterilised with alcohol. A pair of disposable Ag/AgCl surface electrodes (FreeEMG 1000; BTS Bioengineering, Milano, Italy) were placed 2 cm apart over SCM (on the third of the way from the suprasternal notch to the mastoid process and SPL (at the middle of the C4 level on the back of the neck, approximately 6–8 cm to the side) [24–26].

Each patient was instructed to use his or her head to perform movements [27]. Two isometric maximum voluntary contractions (MVC) used for EMG normalized were performed first in the following order: slight head flexion in the supine position for SCM and slight extension of the head in the prone position for SPL with manual resistance and verbal encouragement. MVCs only measured once to prevent fatigue and muscle strain in the participants [28].One-minute rest was performed between two contractions and 3 minutes rest was provided before conducting measurements of EMG [23,29].

**Fig 1:**
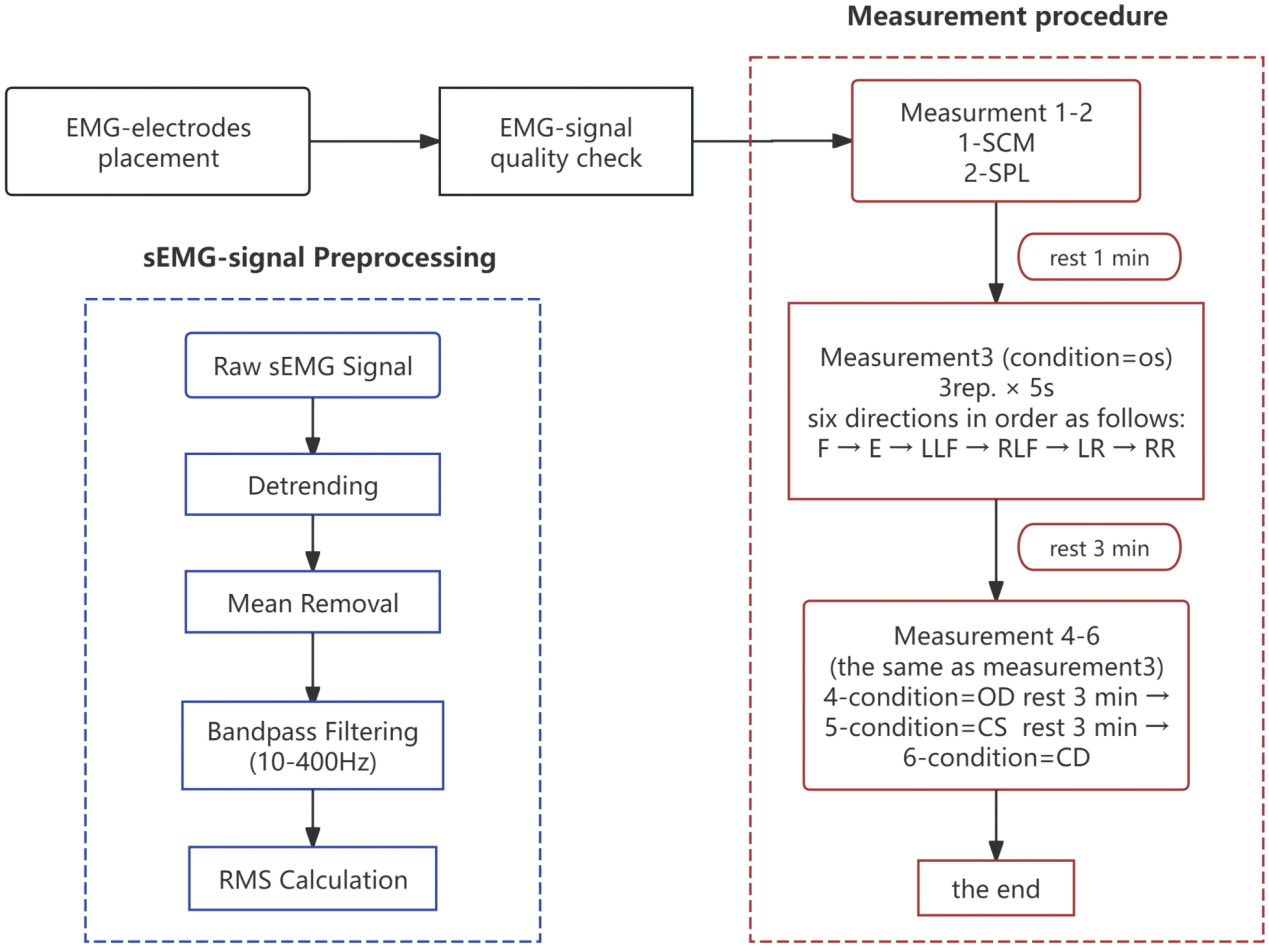
The procedure of measurement and sEMG-signal preprocessing. MVC = maximum voluntary contraction, SCM- sternocleidomastoid, SPL- splenius capitis, E-extension, F-flexion, LR-left rotation, RR-right rotation, LLF-left lateral flexion, RLF-right lateral flexion. CD = eyes-closed and dynamic surface, CS = eyes-closed and stable surface, OD = eyes-open and dynamic surface, OS = eyes-open and stable surface.

For EMG tests which asked patients to exert force while maintaining balance while avoiding severe neck pain under four conditions in order: (1) eyes-open-stable-surface (OS), (2) eyes-closed-stable-surface (CS), (3) eyes-open-dynamic- surface (OD) and (4) eyes-closed-dynamic-surface(CD). On the stable-surface, each participant was required to sit on a chair with the body upright and hip and knee flexed at 90°. On the dynamic-surface, each participant was instructed to sit on a Bobath ball with an upright body with no feet touching the ball. Under each evaluation condition, EMG was measured in six directions in order: flexion, extension, left lateral flexion, right lateral flexion, left rotation and right rotation. Every situation was tested three times with five seconds’ contraction at the same therapist’s manual resistance [30]. A three-minute rest period after each condition was designed to prevent the effects of muscle fatigue. The procedure is illustrated in Fig 1.

### Data analysis

The signals were processed using a root mean square (RMS) algorithm applied to a fixed, non-overlapping window of 3 seconds (equivalent to 1500 samples at our 500 Hz sampling rate), which was extracted from the stable middle portion of each recording.

For each EMG tests, the average measure of three trials was obtained for each situation. Corresponding muscles’ RMS values were normalized for each direction, calculated as the relative EMG level [31]. The mean activation of the bilateral SCM was for flexion, and that of bilateral SPL was for extension [32]. The ipsilateral SCM’s activation corresponded to ipsilateral lateral flexion. The mean activation of ipsilateral SPL and ipsilateral SCM was considered rotation’s target EMG [33]. Then, the target EMG value of each situation was divided by the EMG value of the MVC of the corresponding muscles (flexion-SCM, extension-SPL) to obtain a percentage of maximal voluntary contraction(%MVC) for normalization. The MVC% were regarded as representative values of muscle activation.

### Statistical analysis

We determined the normal distribution of data by the Shapiro-Wilk test (p > 0.05). Two-way repeated measure analysis of variance (ANOVA) was used to analysis main effects and interactions of two within-subject factors (visual: eyes open or closed; stability: surface stable or dynamic) for each direction. If interaction effects are significant, conduct simple effect analyses using Bonferroni-corrected paired t-tests; otherwise, report main effects directly.The data were processed using SPSS version 27 (IBM Corp., Armonk, NY, USA).

## Result

### Characteristics of the participants

A total of 39 patients with CNSNP participated in this study: 14 males and 25 females. The characteristic data of the participants are shown in Table 1. The mean age was 22.26 ± 4.23, and all the experienced neck stiffness. The average sedentary time of the participants reached 7.51 ± 1.96. The NDI and current NRS scores were 10.23 ± 4.26 and 3.92 ± 1.55, respectively. The highest NRS score reached 6.29 ± 1.79.

**Table 1:** Characteristics of the thirty-nine participants.

Variable	Mean±SD
Age(Years)	22.26 ± 4.23
Height(cm)	167.5 ± 8.26
Weight(kg)	60.9 ± 11.33
BMI(kg/m2)	21.55 ± 2.51
Sedentary time(Hours)	7.51 ± 1.96
**NRS**	
Right now	3.92 ± 1.55
The most painful	6.29 ± 1.79
Least painful	1.55 ± 1.65
NDI(points)	10.23 ± 4.26
SAS(points)	44.7436 ± 12.01
SDS(points)	44.7439 ± 12.05
TSK(points)	40.46 ± 6.02

NRS = Numerical Rating scale, NDI = Neck Disability Index, SAS = Self-Rating Anxiety Scale, SDS = Self-Rating Depression Scale, TSK = Tampa Scale of Kinesiophobia.

### Two-factor repeated measures ANOVA

**Fig 2:**
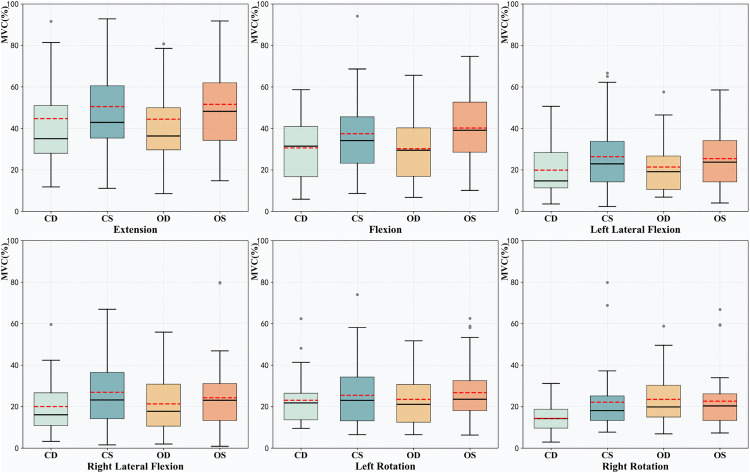
The MVC% of each situation. The red dashed line in the figure represents the mean, while the solid black line represents the median. MVC% = the percentage of maximum voluntary contraction, CD = eyes-closed and dynamic surface, CS = eyes-closed and stable surface, OD = eyes-open and dynamic surface, OS = eyes-open and stable surface.

The MVC% of each situation were shown in Fig 2.

The results of the two-factorrepeated measures ANOVA revealed significant main effect of stability (surface stable or dynamic) for four directions—flexion (F = 21.341, p < 0.001), left lateral flexion (F = 11.016, p = 0.002), right lateral flexion (F = 13.349, p < 0.001), and left rotation (F = 5.068, p = 0.030), without significant interaction effect. For extension, the stability effect marginally reached significance at p = 0.050 (F(1,38)=4.098, p = 0.050, partial η² = 0.0256). Given the significance of stability effect in the remaining directions, a corresponding main effect analysis was performed for this direction, too. The results of the corresponding main effects analysis are shown in the Table 2.

**Table 2:** The results of the two-factor repeated measures ANOVA.

Directions	Main effect				Interaction effect	
	visual		stability		visual * stability	
	F-Ratio	p-value	F-Ratio	p-value	F-Ratio	p-value
**E**	0.024	0.878	4.098	0.050	0.169	0.683
**F**	0.645	0.427	21.341	<0.001**	2.116	0.154
**LLF**	0.031	0.862	11.016	0.002*	1.669	0.204
**RLF**	0.244	0.624	13.349	<0.001**	1.880	0.178
**LR**	0.681	0.415	5.068	0.030*	0.198	0.659
**RR**	10.633	0.002*	5.228	0.028*	7.435	0.010*

E-extension, F-flexion, LLF-left lateral flexion, RLF-right lateral flexion, RR- right rotation. * stands for p < 0.05, ** stands for p < 0.01.

### Main effects

Stability main effect analysis showed significantly higher muscle activation in the stable surface than in the dynamic surface: extension (t = 2.570, p = 0.012), flexion (t = 5.607, p < 0.001), left lateral flexion (t = 4.047, p = 0.001), right lateral flexion (t = 3.568, p < 0.001) and left rotation (t = 2.457, p = 0.0163). The result show in Table 3.

**Table 3:** The results of main effect analysis and mean between-condition differences (95% CI).

Direction	Comparison	Mean (95%CI)	t	p-value
**Extension**	S-D	6.49 (1.46—11.52)	2.570	0.012*
**Flexion**	S-D	8.39 (5.41—11.37)	5.607	<0.001**
**Left Lateral Flexion**	S-D	5.24 (2.67—7.83)	4.047	0.001**
**Right Lateral Flexion**	S-D	4.94 (2.18—7.70)	3.568	<0.001**
**Left Rotation**	S-D	2.78 (0.53—5.02)	2.457	0.0163*

S = stable, D = dynamic. * stands for p < 0.05, ** stands for p < 0.01.

### Simple effect analysis

A significant interaction effect was observed in right rotation (F = 7.435, p = 0.010), main effect of visual (F = 10.633, p = 0.002) and stability (F = 5.228, p = 0.028) were also observed. A simple effect analysis was conducted, yielding a Bonferroni-corrected p-value of 0.0083. The result show in Table 4. Three groups revealed significant differences: i) stable surface with higher muscle recruitment than that in the dynamic surface when the eyes were closed (CS-CD, t = 3.174, p = 0.003); ii)eyes-open condition has significantly greater muscle activation than that in the eyes-closed condition with the dynamic surface (OD-CD,t = 4.782, p < 0.001); iii) eyes-open-stable-surface condition with higher muscle recruitment than that in eyes-closed-dynamic-surface(OS-CD, t = 4.131, p < 0.001). It is worth noting that significant differences are only observed when compared other conditions with CD.

**Table 4:** The results of simple effect analysis of right rotation and mean between-condition differences (95% CI).

Direction	Comparison	Mean (95%CI)	t	p-value
**Right Rotation**	CS-CD	7.82 (2.83—12.82)	3.174	0.003*
	OS-OD	−0.84(−4.70—3.02)	−0.442	0.6608
	OD-CD	9.18(5.29—13.06)	4.782	<0.001**
	OS-CS	0.51(−4.36—5.38)	0.212	0.8333
	OS-CD	8.33(4.25—12.42)	4.131	0.0002**
	CS-OD	−1.35(−5.89—3.18)	−0.605	0.549

Simple effect analysis results, using the Bonferroni corrected p-value of 0.0083.

CD = eye-closed and dynamic surface, CS = eye-closed and stable surface, OD = eye-open and dynamic surface, OS = eye-open and stable surface. * stands for p < 0.0083.

## Discussion

This study determined the effect of visual and support surface stability (which primarily interferes with lower limb proprioceptive input) on seated dynamic balance in participants with CNSNP by altering sensory input during six-directional head resistance exercises, using sEMG signals normalized to MVC% as an indicator. The overall results revealed that the absence of support surface proprioceptive input significantly impaired seated dynamic balance in patients with CNSNP, manifested as a significant decrease in corresponding neck muscles’ MVC%. The presence or absence of visual input did not produce a significant effect, reflecting postural control is more dependent on altered proprioceptive input.

In all six directions’ comparison of stability, a significant higher degree of MVC% were observed in stable surface than in dynamic one, suggesting that proprioceptive inputs were of vital importance for maintaining balance and performing muscle recruitment in the dynamic surface than in the stable surface. Participants needed to expend more effort to maintain balance to perform muscle recruitment in a dynamic surface than in a stable one. Neck proprioception impairment has been observed in patients with CNSNP [34]. Former finding indicated that patients with chronic neck pain compensated impaired neck proprioception more by lower limb proprioception than by other sensory inputs [13]. Study of sensory reweighting of postural control [35] found the compensatory strategies seem to lie within the proprioceptive system, there is no way for impaired neck proprioception to be compensated by overemphasizing other senses in CNSNP patients [36]. These findings also suggests that after the supporting surface proprioceptive input is cut off, the compensatory inputs (including vestibular and visual) are not enough to enable better muscle recruitment while maintaining balance. The same result was demonstrated in another group [37]. The comparison of stability also implies that the clinical treatment of CNSNP can be performed under more complex sensory input conditions, and that training in an unstable surface can help CNSNP patients perform better in sitting balance. However, a cross-sectional study indicated that individuals with neck pain can overcome their impaired proprioception by using their vision [38]. This may be related to the fact that their subjects were cleaners who required high postural demands.

There is insufficient evidence in the experiment to demonstrate the importance of visual input. In most comparisons, there were no significant effect of visual inputs nevertheless the surface stable or dynamic. The finding is in line with previous studies of Winter’s [39] experiment, who used body sway as an indicator, concluding that the visual system had no effect on postural balance. They did not observe significant differences between the eyes-open and closed results at 100% of the width of the stance. The same conclusion was also made by Hiengkaew [40], who examined seated balance and visual vertical perception in patients with neck pain and healthy individuals, demonstrated that seated postural balance was impaired in patients with neck pain, but the impairment was not related to the patients’ visual vertical perception [41]. The compensatory capacity of other systems is sufficient to maintain postural stability in the absence of visual inputs and it may be related to the relatively young age of subjects who participated in the present experiment. Ruhe [41,42] indicated the importance of visual inputs in balance movements increases with age. The participants in our experiment were all under 40 years old and relied more on proprioception in balance movements. Moreover, a seated position was used in this experiment, reducing difficulty in resistance movement to some extent and reducing the importance of visual input.

However, simple analysis in right rotation indicated 3 significant different appeared in comparison of CD and other three conditions. Among these three comparison, one demonstrated the importance of visual input—in dynamic surface there has a higher MVC% in eyes open than in eyes closed. This may associate with situational specificity effects of visual input which was found by Okayama [43]. There has a complex relationship between visual perception and postural stability, which exert considerable effects only under specific conditions. Meanwhile, a study [44] indicates that visual information modulated the threshold for movement-evoked pain, suggesting visual input may play a more significant role in regulating pain.

Our findings of impaired dynamic seated balance, particularly under the dynamic surface condition, can be explained from a fundamental point of view. Central nervous system dynamically and selectively adjusts the relative contributions of sensory inputs in order to maintain balance. Chronic pain can affect body perception at the central level by causing the somatosensory cortex to rearrange, which can alter feedforward and feedback mechanisms [45]. Neck pain also has been shown to impact cortico-cerebellar processing and sensorimotor integration [46], an altered proprioceptive input from neck vibration impacts cerebellar pathways. A research study also indicates that individuals with neck pain rely more on feedback loops which dependent on proprioception [47].

Our research has strengths and limitations. Few trials have compared patients with CNSNP in dynamic and stable conditions with different seated balance and explored differences in sensorimotor function, focusing on young adults aged 18–40 years. For the first time, we used the concept of dynamic seated balance, which is a prerequisite for accomplishing activities of daily living, such as eating, dressing and transferring. For example, forward leaning of the trunk when reaching for an object requires dynamic adjustment of the center of gravity to prevent falls, and patients with inadequate dynamic balance are prone to lose balance during sit-to-stand transitions [40]. This condition increases the risk of fracture especially in osteoporotic patients. Our primary participants were college students, which may limit generalisability to other populations in this age group. We performed EMG measurements and used maximal contraction, which may lead to discomfort, potential injury and risk of delayed muscle soreness [28]. Another primary limitation of this study is the lack of a healthy control group. Therefore, while our findings of altered muscle recruitment on an unstable surface align with the theory of sensory reweighting in CNSNP, future case-control studies are necessary to confirm the specificity of these patterns to the patient population.

## Conclusion

In patients with CNSNP, lower limb proprioceptive input plays a greater role than visual inputs in maintaining balance. Support surface proprioceptive inputs considerably affected myoelectric activation while maintaining balance, whereas visual inputs did not. It is worthwhile to perform more complex sensory input conditions in the clinical treatment of CNSNP, training in an unstable surface can help patients with CNSNP to exercise and perform better in the daily life. Future studies may also conduct subgroup analyses by grouping patients according to different NRS scores.

## Supporting information

S1 Fig Data collection process. (TIF)
